# Major liver resection results in early exacerbation of insulin resistance, and may be a risk factor of developing overt diabetes in the future

**DOI:** 10.1007/s00595-012-0268-8

**Published:** 2012-07-25

**Authors:** Adam Durczynski, Janusz Strzelczyk, Katarzyna Wojciechowska-Durczynska, Anna Borkowska, Piotr Hogendorf, Dariusz Szymanski, Justyna Chalubinska, Leszek Czupryniak

**Affiliations:** 1 Department of General and Transplant Surgery, Barlicki University Hospital, Medical University of Lodz, Kopcinskiego Street 22, 90-153 Lodz, Poland; 2 Department of Endocrinology and Metabolic Diseases, Polish Mother’s Memorial Hospital, Medical University of Lodz, Lodz, Poland; 3 Department of Internal Medicine and Diabetology, Barlicki University Hospital, Medical University of Lodz, Lodz, Poland; 4 Department of Radiation Oncology, Kopernik Memorial Hospital, Medical University of Lodz, Lodz, Poland

**Keywords:** Liver tumor, Hemihepatecomy, Glucose homoeostasis

## Abstract

**Purpose:**

This single center prospective cohort study evaluated the influence of hemihepatectomy on glucose homeostasis.

**Methods:**

The study included 30 patients undergoing hemihepatectomy. All patients underwent an oral 75 g glucose tolerance test before (baseline), 1 week and 1 month after the surgery. Plasma glucose, insulin and glucagon were measured in the OGTT samples, and the HOMA index was calculated. The fasting levels of interleukin 6 and 1β, tumor necrosis factor and adiponectin were assessed.

**Results:**

The fasting plasma and 120-min post-challenge mean glucose level increased during the study from 89.6 to 103.5 mg/dl (by 15.5 %) and from 136.4 to 162.2 (by 18.9 %; *p* = 0.51), respectively, accompanied by an increase in fasting glucagon (from 3.2 to 5.9 ng/mL; *p* = 0.043) and insulin (from 14.6 to 19.3 IU/mL) and by a decrease in plasma insulin at 60 min of OGTT (*p* = 0.34). An increase of IL-6 (*p* = 0.015) and TNF (from 49.7 to 53 pg/mL), and decrease of plasma APO (7658 to 5152 ng/mL) and exacerbation of insulin resistance (*p* = 0.007) were noted.

**Conclusion:**

Hemihepatectomy resulted in moderate disturbances in glucose homeostasis, in a majority of patients that was likely to be of minor clinical relevance. However, the patients might be at higher risk of developing overt diabetes following long-term survival.

## Introduction

Liver diseases increase the risk of developing glucose intolerance [1], since hepatocytes play an important role in maintaining narrow range of the normal plasma glucose concentration. An increasing number of patients with liver tumors are treated with hemihepatectomy [2], as it can provide long-term survival or sometimes cure in the majority of patients with primary and metastatic malignant tumors [3, 4]. As a result, the number of survivors has increased worldwide, and many new clinical questions regarding metabolic disorders have arisen in these patients.

The effect of hemihepatectomy in humans on glucose homeostasis remains mostly unknown. The aim of this study was to assess the influence of hemihepatectomy performed due to liver tumor on glucose homeostasis in non-cirrhotic and non-diabetic patients.

## Methods

This single center prospective cohort study enrolled patients hospitalized in the Department of General and Transplant Surgery of Medical University in Lodz, Poland, from May 2008 to June 2009 that underwent right or left hemihepatectomy due to a resectable solitary tumor.

All patients provided written informed consent and underwent an oral glucose tolerance test (OGTT) according to World Health Organization protocol [5] before (baseline), 1 week and 1 month after the surgery.

The subjects with cirrhosis and glucose intolerance or frank diabetes or taking medications affecting plasma glucose were excluded from the study.

Although, glycosylated hemoglobin A1c (HbA1c) might be useful in assessing glucose metabolism in diabetic patients preoperatively, it is difficult to evaluate glucose homeostasis using only the HbA1c level in subjects with liver disease [6], thus it was not analyzed.

Surgery was performed through a right subcostal incision. The extent of local disease and extrahepatic metastases was assessed during surgery, and intraoperative ultrasound was performed to assess the relationship between the tumor and the vascular structures. Hepatic vascular inflow was occluded using the Pringle maneuver (mean time 17 ± 2.3 min) during liver parenchymal transection with a clamp-crushing method. Major vascular branches and bile ducts were secured intrahepatically with clamps and ligated. Hemostasis was performed on the parenchymal surface of liver remnant. Intra-abdominal drainage was used in all patients.

Plasma glucose (Bio Systems Glucose Assay Kit), insulin (DakoCytomation Insulin ELISA Kit) and glucagon (Phoenix Pharmaceuticals Inc. Human Glucagon ELISA Kit) were measured at 0, 60, and 120 min after ingestion of 75 g glucose, and HOMA index was calculated (fasting plasma insulin × fasting plasma glucose/22.5).

Moreover, fasting plasma adiponectin (APO), tumor necrosis factor (TNF), interleukin IL-1β and IL-6 (selected liver regeneration promoting cytokines affecting glucose metabolism; all assays performed with the use of R&D ELISA commercial kits) were evaluated.

Perioperative routine glycemic control of these patients included blood glucose measurements before induction of anesthesia, during surgery, and two hourly in the intensive care unit for 48 h postoperatively.

Statistical analyses were carried out using the STATISTICA 8.0 software package with the level of statistical significance at *p* < 0.05. Friedman’s ANOVA for repeated measures was used to analyze differences in values between study time-points. A post hoc Wilcoxon test with Bonferroni correction was used when statistically significant effects were observed. Non-sphericity of data was verified by the Greenhouse-Geisser and Huhn-Feldt tests; Levene test of homogeneity variances was applied. All data are given in the text, tables and figures as the means plus standard deviations (±SD).

## Results

The study group included 30 patients (mean age 58.0 ± 12.2 years, body mass index BMI 26.5 ± 5.0 kg/m^2^). All patients had normal liver enzyme levels preoperatively, tumors were localized in the right in 20 cases and in the left hepatic lobe in 10, and appropriate hemihepatectomies were performed (Table 1). The histopathological examination revealed 13 metastatic colorectal and 9 hepatocellular cancers, 5 adenomas and 3 hemangiomas.

**Table 1:** Patient and tumor characteristics, operative details and outcomes

Hepatitic status, *n*	
Hepatitis B Cartier	0
Hepatitis C Cartier	0
Liver cirrhosis, *n*	0
Liver functional status, *n*	
Child-Pugh Grade A	30
Child Pugh Grade B	0
Comorbidities, *n*	
Hypertension	13
Ischemic heart disease	5
Preoperative laboratory tests	
Hemoglobin (g/dL)	13.1 ± 2.2
Platelet count (10^6^/mL)	194 ± 33
Aspartate aminotransferases (U/L)	19.5 ± 3.5
Alanine aminotransferases (U/L)	16.0 ± 5
Bilirubin (md/dL)	0.84 ± 0.5
Prothrombin time (s)	14.2 ± 0.8
Albumin (g/L)	39.1 ± 13.2
Creatinine(μmol/L)	59.2 ± 22
Indications for resection, *n*	
Hepatocellular carcinoma	9
Metastases of colorectal cancer	13
Hemangioma	5
Hepatic adenoma	3
Tumor nodules, *n*	1
Tumor size (cm)	7.5 ± 5.2
Tumor volume (cm^3)^	110 ± 25
Types of liver resections, *n*	
Right hemihepatectomy	20
Left hemihepatectomy	10
Operative time (min)	135 ± 45
Intraoperative blood loss (mL)	430 ± 140
Transfusion requirements	
Patients transfused, *n*	9
Packets red cells (U)	2.6 ± 0.8
Patients with complications	4
Type of complication, *n*	
Hepatic insufficiency	0
Hemorrhage	0
Bile leak	0
Atrial fibrillation	1
Superficial wound infection	2
Pulmonary thrombosis	1
Reoperation	0
Postoperative hospital stay (days)	10.1 ± 3.1

Previous studies reported non-significant weight loss following hepatic resection [7], thus BMI alterations (26.5 ± 5.0 (baseline), 25.5 ± 3.5 and 25 ± 4.5, respectively) were not further co-analyzed. Study time-points were precisely predefined to exclude the influence of postoperative malnutrition on glucose metabolism; diet-related bias should be excluded.

The fasting plasma glucose level increased during the study from 89.6 to 103.5 mg/dl (by 15.5 %), while post-challenge glucose remained relatively constant at 60 min and rose from 136.4 to 162.2 at 120 min (by 18.9 %; *p* = 0.51).

**Fig. 1:**
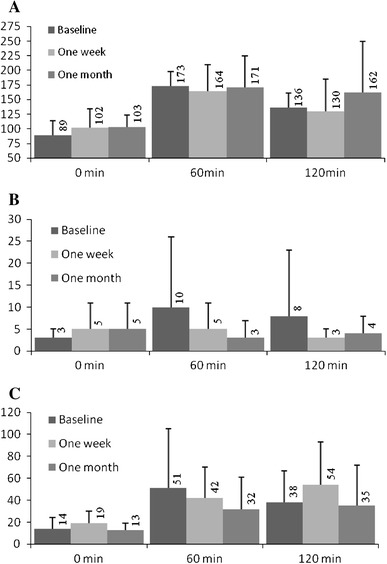
**a** Glucose (mg/dL), **b** Glucagon (ng/mL) and **c** Insulin (IU/mL) mean plasma concentrations in oral glucose tolerance test before (baseline), 1 week and 1 month after hemihepatectomy. Changes of glucose and insulin were non significant (*p* = 0.51 and *p* = 0.34, respectively); glucagonemia increased 1 month postoperatively in comparison to baseline (*p* = 0.043)

**Fig. 2:**
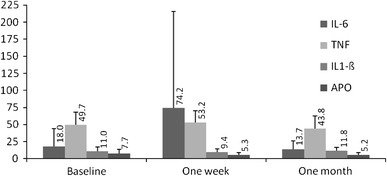
Mean fasting plasma concentrations of APO (pg/mL), IL-6 (pg/mL), TNF (pg/mL) and IL-1β (pg/mL) before (baseline), 1 week and 1 month postoperatively. IL-6 increased 1 week postoperatively (*p* = 0.015) and decreased 1 month after surgery (*p* = 0.002). Changes of APO, TNF and IL-1β were non significant (*p* = 0.18, *p* = 0.22, and *p* = 0.14, respectively). HOMA index (fasting plasma insulin × fasting plasma glucose/22.5) was calculated (3.38 ± 3.3, 4.74 ± 3.13, 3.28 ± 1.85, respectively). The HOMA index was significantly higher 1 week postoperatively in comparison to baseline (*p* = 0.007)

The changes in plasma glucose were accompanied by an increase in fasting glucagon and insulin and by a decrease in plasma insulin at 60 of OGTT (Fig. 1). An increase of IL-6 (*p* = 0.015) and TNF (from 49.7 to 53 pg/mL), and a decrease of plasma APO (7658–5152 ng/ml, respectively) were noted after hemihepatectomy, the surgery also resulted in exacerbation of insulin resistance evaluated by HOMA index (*p* = 0.007; Fig. 2).

## Discussion

Major abdominal surgery may cause disturbances marked in glucose homeostasis including stress hyperglycemia [8], that should be diagnosed in all patients with blood glucose exceeding 110 mg/dL [9]. The current study found that the increase of fasting plasma glucose was moderate (Fig. 1), but nevertheless still might have been clinically relevant, since it was showed that even moderate postoperative elevation of plasma glucose may lead to perioperative complications [10, 11].

The degree of insulin resistance is related to severity of the operation [8]. The increased 1 week HOMA index and decreased adiponectin (Fig. 2) suggest that marked insulin resistance develops soon after hemihepatectomy, which is a typical body reaction to a severe health condition [12]. Insulin resistance leads to hyperinsulinemia, which might also have been stimulated in the studied group by the increase in plasma glucagon.

A 1-month reduction of the postoperative acute phase response led to a decrease in IL-6 and insulin resistance. The remaining long-term hyperglucagonemia (Fig. 1) may be partly due to reduced hepatic fractional extraction [13], but also enhanced glucose requirements for liver regeneration. Therefore, hyperglucagonemia with decreased adiponectin is likely to be involved in maintaining indispensable fasting hyperglycemia during the late recovery period. Interestingly, these changes in glucose metabolism seen after hemihepatectomy are similar to those developing over years in patients with type 2 diabetes mellitus, and may be encountered more frequently, as perioperative outcomes and long-term survival of these oncologic patients have significantly improved [3, 4].

In general, the current study demonstrated that insulin resistance may be exacerbated following hemihepatectomy (Fig. 2). The precise mechanism of impaired insulin activity among patients with liver failure has not been described [14, 15]. Proinflammatory interleukins may participate in the development of insulin resistance either by suppressing insulin receptors tyrosine kinase activity or reduction of transmembrane glucose transporters expression. Therefore, increased plasma IL-6 and TNF after liver resection (Fig. 2) may trigger disturbances in glucose homeostasis during the early postoperative period.

The glucose clamp method continues to be the reference standard to quantify insulin sensitivity [16]; however, it is complex, time consuming and expensive to simply test fasting blood samples. Therefore, the glucose clamp method is not used in large clinical studies or routine clinical application where simpler methods are required. The current study used the surrogate HOMA index, which is among the best and most extensively validated.

Intensive insulin therapy was avoided in the current patients since it is related to high incidence of life-threatening hypoglycemia [17]. However, accurate computer-assisted blood glucose control is used to reduce the incidence of hypoglycemia in patients after hemihepatectomy; although it is still not very accessible [18].

IL-1β that was shown to influence glucose utilization and insulin secretion [19] remained stable during study (Fig. 2). However, postoperative elevation of this acute phase immune response interleukin and its normalization within 7 days postoperatively cannot be excluded.

Past studies evaluated the clinical consequences of the activity of various biochemical adiponectins on glucose homeostasis, as well [20]. Likewise, adiponectin plasma concentrations were analyzed in the current study and a tendency toward a stable decrease of this adipocytokine concentration was observed in patients following hemihepatectomy (Fig. 2). A low plasma adiponectin level may be valid predictive factor of diabetes development in the future and may be related with reduction of hepatocytes insulin sensitivity [20], and this phenomenon may co-trigger development of hyperglycemia. Furthermore, there is elevation of plasma adiponectin level in patients with liver failure [21], yet no similar elevation was observed in the current series, thus suggesting considerable liver clearance capacity of this adipocytokine.

There were discrepancies in the concentration of early endogenous mediators of inflammation, only the elevation of IL-6 was statistically significant. The serum levels of IL-6 remained elevated for several days after elective surgeries implying its persistent production, although no changes of plasma IL-1 and TNF were observed [22]. The higher IL-6 levels after hemihepatectomy may be due to the loss of IL-6 uptake as a result of a smaller functional remnant liver [23]. Furthermore, some authors indicate that IL-6 suppresses IL-1β and TNF production [24].

Although the current study design lacked a control group including other abdominal surgeries, all data on each patient were analyzed in comparison to the baseline. However, further long-term controlled comparison studies in large patient groups are warranted to confirm the current results.

In conclusion, hemihepatectomy in subjects with liver tumors results in a moderate disturbance in glucose homeostasis, which should be of minor clinical relevance in the majority of patients. Still, the subjects with risk factors for glucose intolerance, e.g., family history of diabetes, overweight, with past gestational diabetes, that are considered for liver surgery should be monitored carefully for changes in their glucose metabolism to minimize the risk of perioperative complications. Moreover, long-term survival might be associated with a higher risk of developing overt diabetes.
